# Hydrokinetic Turbine Effects on Fish Swimming Behaviour

**DOI:** 10.1371/journal.pone.0084141

**Published:** 2013-12-17

**Authors:** Linus Hammar, Sandra Andersson, Linda Eggertsen, Johan Haglund, Martin Gullström, Jimmy Ehnberg, Sverker Molander

**Affiliations:** 1 Department of Energy and Environment, Chalmers University of Technology, Gothenburg, Sweden; 2 Marine Monitoring AB, Lysekil, Sweden; 3 Department of Ecology, Environment and Plant Sciences, Stockholm University, Stockholm, Sweden; Pacific Northwest National Laboratory, United States of America

## Abstract

Hydrokinetic turbines, targeting the kinetic energy of fast-flowing currents, are under development with some turbines already deployed at ocean sites around the world. It remains virtually unknown as to how these technologies affect fish, and rotor collisions have been postulated as a major concern. In this study the effects of a vertical axis hydrokinetic rotor with rotational speeds up to 70 rpm were tested on the swimming patterns of naturally occurring fish in a subtropical tidal channel. Fish movements were recorded with and without the rotor in place. Results showed that no fish collided with the rotor and only a few specimens passed through rotor blades. Overall, fish reduced their movements through the area when the rotor was present. This deterrent effect on fish increased with current speed. Fish that passed the rotor avoided the near-field, about 0.3 m from the rotor for benthic reef fish. Large predatory fish were particularly cautious of the rotor and never moved closer than 1.7 m in current speeds above 0.6 ms^-1^. The effects of the rotor differed among taxa and feeding guilds and it is suggested that fish boldness and body shape influenced responses. In conclusion, the tested hydrokinetic turbine rotor proved non-hazardous to fish during the investigated conditions. However, the results indicate that arrays comprising multiple turbines may restrict fish movements, particularly for large species, with possible effects on habitat connectivity if migration routes are exploited. Arrays of the investigated turbine type and comparable systems should therefore be designed with gaps of several metres width to allow large fish to pass through. In combination with further research the insights from this study can be used for guiding the design of hydrokinetic turbine arrays where needed, so preventing ecological impacts.

## Introduction

Climate change mitigation and the increasing demand for renewable energy have revived the development of ocean energy systems, including open flow hydrokinetic turbines targeting the energy of fast-flowing currents [1,2]. Existing hydrokinetic turbines are of various sizes and rely on several different energy capture principles [3]. Most of these turbines are still at pre-commercial stage but a few are deployed at full scale [4]. Given that the accelerating technical progress leads to cost reductions, large arrays of hydrokinetic turbines are likely to be installed for power generation in fast-flowing (>1 ms^-1^) estuaries, tidal channels and around coastal bends [1,5,6].

Hydrokinetic turbines extract energy through horizontal- or vertical-axis rotors with blades moving rapidly through the water. Potential collisions between the rotor and marine fauna has repeatedly been pointed out as an environmental concern associated with high uncertainty (e.g. Gill [7]; Wilson et al. [8]; Boehlert & Gill [9], Frid et al. [10]). A few reports of fish monitoring at deployed turbines [11-13] and a controlled fish-turbine experiment [14,15] have recently become available. These important studies indicate that impact is low, but few species are covered, and the effects on fish swimming behaviour are not covered in detail. Also, none of these studies have been published in the scientific literature.

More detailed studies are therefore needed to increase the understanding of rotor effects on fish [16] and to improve modelling of collision risks [17]. If some species, groups or life stages of fish are found to be sensitive to hydrokinetic turbines, as has been the case among conventional hydropower and cooling water intakes [18-20], long-term ecological consequences may occur. For instance, even if fish avoid collision the avoidance zone might be larger than the actual rotor and so multiple turbine systems may hinder fish migration [21]. Such migratory restrictions may ultimately affect patterns of seascape connectivity, which is of high importance in both tropical [22] and temperate coastal ecosystems [23].

Hydrokinetic turbines all target fast-flowing currents but different devices are designed for different depths and conditions, so potential sites are found in a wide range of locations [6], potentially affecting a large number of fish species. As most turbines occupy mid-water depths, pelagic and semi-pelagic species may be of highest concern [8]. However, small turbines can also be positioned in shallow water adjacent to land [3,24,25] making benthic species of interest as well.

Different fish species are distinguished by physiological and behavioural traits relevant for their response to a moving turbine rotor. Fish swim in different ways, some being specialized for cruising and sprinting, while others, such as many reef fishes, are adapted for high manoeuvrability [26,27]. Fish swimming speed is generally highest among pelagic predators [27] and is positively correlated to body length with large individuals moving faster [28]. However, speed relative to body size decreases with fish size [29] and acceleration can be faster for small fish [27]. Strikes from rotor blades may therefore be more difficult to avoid for large individuals, as has also been suggested from collision risk modelling [8,17]. Moreover, the strong currents at hydrokinetic turbine sites may challenge the manoeuvrability, particularly of fish with less streamlined body shape. Any of these morphological traits (swimming style, size and body shape) may thus affect the response to hydrokinetic turbines. 

But it is not just swimming performance that determines the ability to avoid collision. Fish would typically benefit from detecting a moving object, like a rotor, at distance. Although turbine noise emissions may be detected by fish, thereby initiating a deterring or attracting response among different species, it is likely that vision is the prime sensor for fish to identify a turbine rotor in strong currents [8,17]. Visual stimuli are highly important for initiating escape response among prey [30], and low-light conditions have been shown to reduce the detection distance to predator stimuli in various species [31,32]. Alongside current velocity, light and turbidity should therefore be important environmental conditions influencing fish response to turbines.

Having detected a rotor, behavioural characteristics such as boldness – the reaction to a situation perceived as dangerous, as defined by Réale et al. [33] – come into play. The level of boldness differs among species and among individuals [34,35] and is likely to affect a fish’s susceptibility to the threat of a rotor. Moreover, it is possible that the response is related to the trophic level of fish, which in turn is linked to its feeding preference.

With detailed understanding about fish response to rotors, technical adjustments can be made to newly developed turbines if necessary. Should energy installations not be hazardous, these may even be perceived as sheltered and therefore preferable habitats for many fish species [36,37]. As was concluded by Inger et al. [37] marine renewable energy has the potential to be both detrimental and beneficial to certain species, but evidence still remains limited.

This study aims at improving the understanding of fish response, in particular swimming behaviour, in relation to hydrokinetic turbines. We investigated daytime effects of a vertical-axis hydrokinetic turbine rotor on numerous reef-associated and pelagic fish species under natural conditions through a field experiment in a subtropical tidal channel. We hypothesized that: (1) the rotor constitutes a hazard to fish, (2) the rotor affects fish swimming behaviour, (3) effects are influenced by changing environmental conditions and (4) effects differ among contrasting fish groups.

## Materials and Methods

### Investigated turbine rotor

A vertical-axis triple-helix reproduction of the Gorlov Helical Turbine was used in the conducted field experiment because of its advanced development stage and due to its wide range of applications, from kilowatt-scale use at remote locations to megawatt-scale multiple turbine arrays [6,38,39]. The Gorlov Helical Turbine has been piloted in several countries and is now deployed at the Uldolmok tidal power station (South Korea) and the Maine Tidal Energy Project (USA). More information can be downloaded from the Gorlov Helical Turbine webpage: http://www.gcktechnology.com/GCK/pg2.html.

The tested rotor had a cylindrical shape of dimensions 1.5×0.7 m, and three twisted 0.1 m wide NACA0020-profiled rotor blades. Being fixed to its foundation by bearings at its base, the rotor was free to spin with the current, using no load or motor. When present, the rotor was always rotating. The rotational speed was correlated to current speed (*r*=0.93, *P*<0.001) and varied from 15 to 70 rpm during the sampling. Like the original design, the rotor was painted in a midnight-blue colour. As will be further discussed, the colouration is likely to have influenced the results.

### Study site

The study was carried out during March–April 2012 in a narrow tidal strait, Ponta Torres, between Inhaca Island and the mainland of Mozambique. Inhaca Island separates the Indian Ocean from the shallow Maputo Bay and moderately strong currents (~1.5 ms^-1^) flow through the strait, following the semidiurnal tides [40]. The location consists of mineral sand bottoms and rocky reefs, sparsely covered by coral. Fishing is prohibited in the area and neighbouring habitats include mangroves, seagrass meadows, algal belts, mudflats, coral reefs and the open ocean, therefore supporting a high abundance and diversity of fish.

The field study took place within the Ponta do Ouro partial Marine Reserve and was approved by the reserve authority Estação de Biologia Marítima da Inhaca, Universidade Eduardo Mondlane. No vertebrates were sampled or injured by the study.

### Experimental design

**Figure 1:**
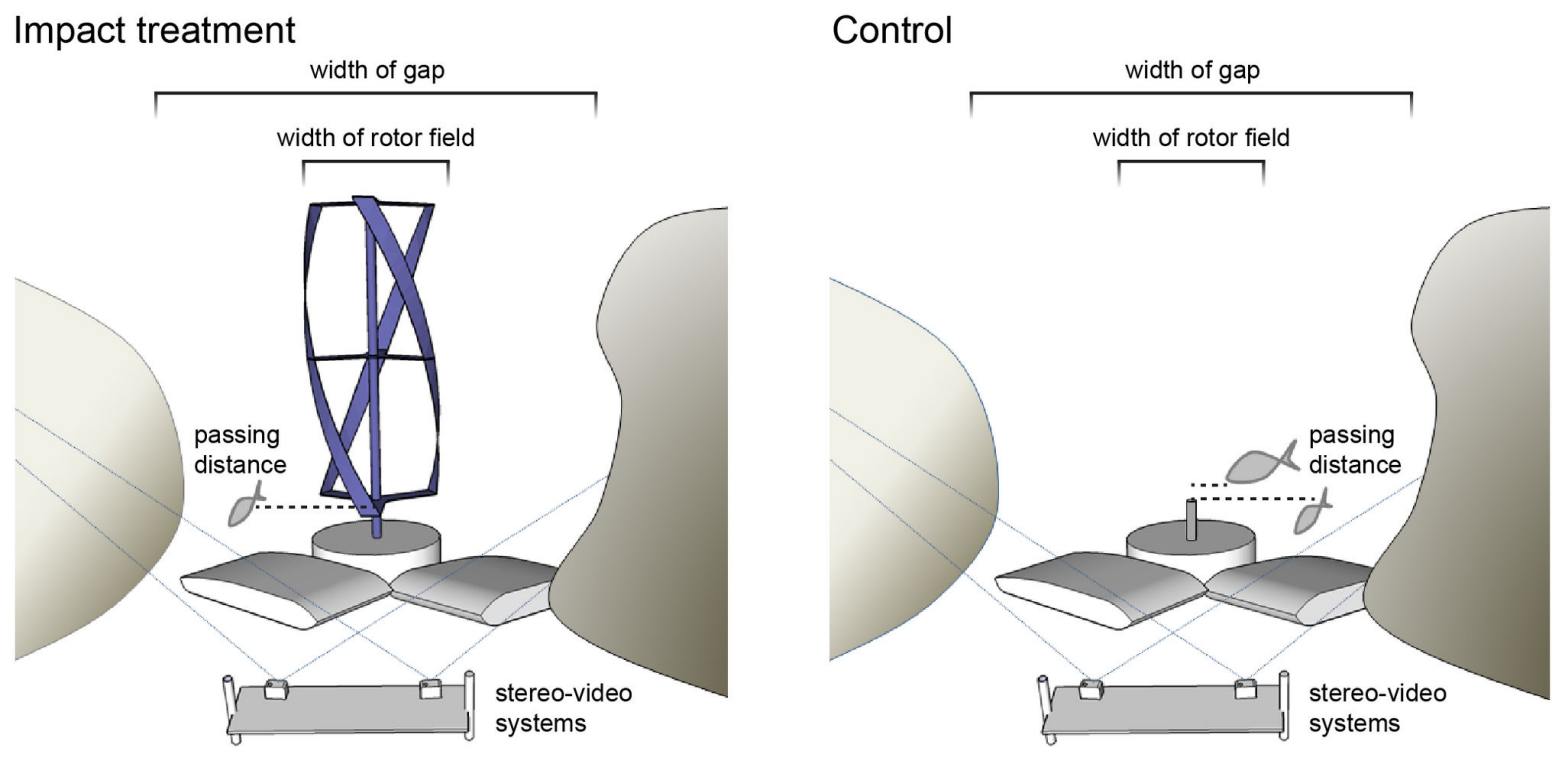
Experimental set-up for impact and control sampling. Fish movements were recorded by remote stereo-video systems and categorized as ‘rotor passages’ if swimming through the rotor field (0.7 m wide) and as ‘gap passages’ if swimming through the gap (2 m wide). The gap included the whole cross-area between the two rock formations, that is, both the rotor field and the space between rotor and rocks. Measurements of fish length and the closest distance (dotted horizontal lines) between passing fish and the rotor centre were computed for fish passing within the camera stereo-field (illustrated as overlapping camera fields of view).

The experiment was set up on a rocky reef bottom at 9 m depth, 15 m from shore. The rotor was positioned in the middle of an approximately 2 m wide opening between rock formations. The full cross-area between the rocks was defined as the ‘gap’ (Figure 1). The cross-area of the rotor (inside the gap) was defined as the ‘rotor field’. Fish could thus choose among three options: (i) pass through the gap within the rotor field, (ii) pass through the gap at either side of the rotor field or (iii) not pass through the gap. Using remote stereo-video systems, fish movements were sampled with and without the rotor installed (impact vs. control). Sampling was carried out using a hierarchical design with four random sampling occasions for control and impact respectively and five replicates within each occasion. 

All sampling was performed in daylight and during ebb currents, ranging from 0.25 to 1.40 ms^-1^. Weather conditions varied among sun, overcast and rain, and wind speeds from 2 to 9 ms^-1^.

### Camera systems and stereo-video sampling

Video recording is considered to be the optimal method for detailed studies of fish behaviour in relation to hydrokinetic turbines [16]. In contrast to single camera systems, stereo-video systems further support quantitative sampling of lengths and distances. The technique implies recording the same object with two synchronized converged cameras – generating highly accurate, impartial and repeatable estimations of its three-dimensional coordinates [41]. We used GoPro^®^ HERO2 cameras in flat-lens underwater housings fixed and calibrated to boards, with 0.8 m base separation and 4° convergence to the centre axis for each camera.

**Figure 2:**
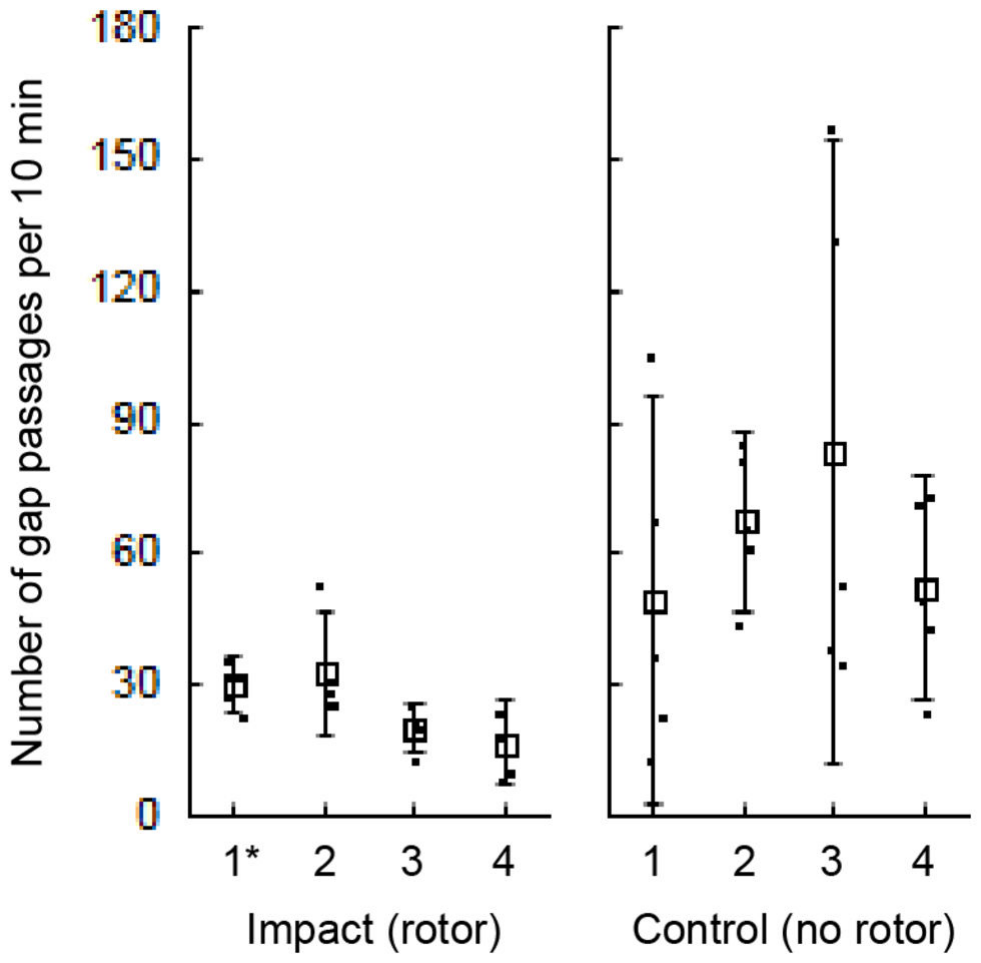
Numbers of counted fish passing through the gap during impact and control sampling. Each sampling occasion comprises a total of 50 min analysed video and the 10 min sample replicates are indicated by dots. Means and 95% confidence intervals are displayed for each of the four different occasions (1–4) at each level of treatment (impact and control). The sampling occasion with use of spare camera systems (positioned above the rotor) is indicated (*).

Deployed by divers, the main video systems were positioned 1.4 m in front of the gap and rotor (viewing along the direction of current, see Figure 1) while spare systems were positioned on the rocks viewing down towards the gap. Recordings from the main systems were used for all but one sampling occasion. The difference in camera positioning between main and spare systems had little effect on fish identification [42] (see Figure 2 for comparison). The stereo-field (the zone allowing for size and distance measurements) was about 1.5 m wide at the centre of the rotor and 6 m wide at 5 m distance. Fish movements at the uppermost part of the rotor were not covered by the field of view.

The video recordings from each sampling occasion were treated according to the following procedures. Video sequences with insufficient visibility or other disturbance were disregarded, along with the first two minutes after the presence of divers potentially having affected fish behaviour [43]. The remaining video sequences were split into 5 min periods separated by 2 min intervals. After analysis, sequential 5 min periods were pooled into samples comprising 10 min recordings in order to reduce data variability. Five such samples were randomly drawn from each sampling occasion and were used as the replicate level of samples. Using a sample unit of 10 min instead of treating each occasion as one independent replicate was motivated by high temporal variation in current speed and water turbidity. In summary, the four sampling occasions, each consisting of five sample replicates of 10 min each, for both the control and the impact treatment, comprise 400 min of analysed video. Two hundred minutes for control and 200 for the treatment.

For all samples, fish identities and movements were extracted, along with environmental variables. Measurements were computed and logged using the EventMeasure (www.seagis.com.au) software. All video analyses were executed by the same observer.

### Fish identity and categorization

Each observed fish was identified to the lowest taxonomic level possible, and genus was used for taxonomic comparisons. Fish were further categorized on the basis of feeding guild, body shape and swimming style. Feeding guild was sorted into browsers (including herbivores and browsers of small invertebrates, e.g. coral polyps), invertebrate feeders and fish/invertebrate feeders based on Froese & Pauly [44]. Body shape was based on Lindsey [26] and included three different types: fusiform (torpedo-shaped body), compressiform (laterally compressed body) and globiform (spherical body). The swimming style categories, determined by body parts engaged in locomotion [27], were subcarangiform (caudal fin and trunk), carangiform (caudal fin), labriform (pectoral fins), balistiform (anal and dorsal fins) and other (including ostraciiform and tetraodontiform fish propelled by undulating movements of caudal or anal and dorsal fins). 

### Fish movement categorization

Fish movements in relation to the gap and the rotor field were categorized as: ‘rotor passage’ (fish passing through the rotor field, that is, through the actual rotor during impact sampling or through the same but empty cross-area during control sampling), ‘gap passage’ (fish passing through the full cross-area between the rocks either through or beside the rotor field, thus the gap passage category also includes rotor passages) or ‘not passing’ (fish only observed behind or in front of the gap). For fish moving within the camera stereo-field ‘total lengths’ of fish and the closest ‘distance’ between fish and the central spindle of the rotor were measured (Figure 1). When the rotor was not present (control samples) this ‘distance’ was measured using a fictive rotor spindle imposed to the video record, guided by the ever-present rotor foundation and validated using the coordinates for the measured objects. The distance between fish and the rotor field (i.e. the edge of the rotor) could then be calculated by subtracting the rotor radius. In addition, distinctive evasive manoeuvres of fish, defined as quick burst swimming away from the rotor [13], were noted. Every fish entering the field of view was counted as a new specimen, even if the same individual returned to the scene. Thereby several individuals are likely to have been counted multiple times. 

A number of large specimens of predator species, mostly kingfishes of the *Caranx* genus, were observed close to the gap but rarely passed through, irrespective of the rotor presence. To examine possible effects on these large fish the distance between fish and rotor field was estimated for all specimens spotted within 2 m of the rotor field.

### Environmental variables

The stereo-video function was also used for estimating tidal current speed and visibility, which are the environmental variables most likely to affect fish behaviour other than temperature [45]. Water temperature, however, was fairly constant throughout the study (26–27°C). Real-time approximations of the current speed were derived from repeated (1 min^-1^) speed measurements of drifting pieces of seagrass. This method was validated through a correlative comparison (*r*=0.96, *P*<0.001) with a Doppler current meter (ALEC Infinity-EM) deployed at the same site before and after sampling. Visibility (basically a measure of water turbidity) was estimated based on the maximum distance at which a medium-sized fish could be distinguished, using 0.5 m intervals from 2 to 6 metres.

### Univariate data analyses

One-factorial non-parametric analysis (Mann-Whitney *U* test) was used to investigate the effect of treatment on rotor passages because fish passages through the actual rotor were very few. The effect of treatment on gap passages could be examined in more detail and to account for effects of important environmental variables we used a two-factor analysis of covariance (ANCOVA) [46] with current speed as covariate and the treatment factor nested in the random factor of occasion. Visibility was excluded from the ANCOVA because of its correlative relation (*r*=-0.54, *P*=0.002) to current speed [46]. To achieve normally distributed data and to meet the assumption of homogeneity of variance, the dependent variable was subjected to a Box–Cox transformation [47]. Homoscedasticity could only be realized by removing two outliers from the control samples (the outliers represented high numbers of gap passages and their removal should lower the effect of treatment). We ran the ANCOVA analysis with and without the outliers and got similar results, presenting the results of the latter. Independencies of variances and means among groups were established through visual examination of plotted standard deviations against means. Linear relationships between each dependent variable and the covariate were confirmed, and we established homogeneity of regression slopes by separate tests for the interactions between each factor and the covariate [46].

Least square linear regression was applied for further examining the influence of environmental variables on fish response to treatment (presence/absence of rotor) and for checking possible relationships between fish length and distance to the rotor during passage. Homogeneity and normal distribution of residuals and model linearity were established by visual inspection of plotted residuals. 

One-factorial analyses targeting effects of treatment on different fish categories and evasion manoeuvres were conducted through non-parametric statistics (Mann–Whitney *U* test and Kruskal–Wallis test with multiple comparisons of mean ranks as post hoc test) or ANOVA when data distributions conformed to the assumptions of the test. Estimations of statistical power were calculated from *t*-test statistics, for α=0.05. Since parametric counterparts of non-parametric tests generally have higher power [48] the *t*-test based power estimates should give an indication of upper level of power. All univariate analyses were computed in STATISTICAv64 (StatSoft Inc.).

### Multivariate data analyses

Effects of treatment on assemblage composition of fish passing through the gap were tested using one-way analysis of similarity (ANOSIM) [49]. Further, taxa with high contributions to dissimilarities between treatment and control samples were identified through similarity of percentages analysis (SIMPER) [50], which is generally a procedure to determine the taxa that contribute most to dissimilarities in community structure among different cluster treatments [49]. Both analyses were based on Bray–Curtis similarity matrices of untransformed abundance data and computed in PRIMERv6 (PRIMER-E Inc.).

## Results

### Main effects of the rotor on fish movements

There was a heavily reduced passage of fish through the rotor field when the rotor was present compared to when it was absent (Mann–Whitney *U* test, *n*=20, *P*<0.001). During control conditions 10.5±2.1 (SE) specimens per 10 min sample moved through the empty rotor field compared to 0.1±0.1 (SE) when the rotor was present. The only two observed specimens inside the spinning rotor were both bluestreak cleaner wrasses (*Labroides dimidiatus* V.) and their presence was associated with low current and rotational speeds (17 rpm).

A total of 1757 gap passages were registered involving specimens from 37 genera. There were significantly more gap passages when the rotor was absent compared to when it was present, whereas there was no significant effect of sampling occasion (Figure 2, Table 1). Moreover, the ANCOVA-analysis showed that the number of gap passages was also significantly affected by current speed (Table 1). Similar results were derived from running the ANCOVA exclusively on fish passing alongside the rotor (i.e. disregarding specimens moving through the rotor field).

**Table 1:** Results of nested ANCOVA on the effects of sampling occasion and treatment on fish gap passages, while controlling for effects of current speed.

**Source of variation**	**d.f.**	**MS**	***F***	***P***
Occasion	3 (3)	0.593 (2.096)	0.342 (0.371)	0.798 (0.779)
Treatment (Occasion)	4 (4)	2.129 (6.890)	18.466 (12.995)	**0.000 (0.000)**
*Covariate*				
Current speed	1 (1)	1.849 (6.736)	16.032 (12.705)	**0.000 (0.001)**
Error	29 (29)	0.115 (0.530)		

The variable under test is the number of fish movements through the gap per 10 min sample. The tested levels of treatment were impact and control, that is, presence and absence of the rotor. Numbers in brackets indicate test results for gap passages with all specimens moving through the rotor field disregarded. Significant effects are indicated in bold.

### Influence of current speed

As current speed was demonstrated to be important, and as visibility was correlated to current speed, the two environmental variables were further examined. A negative relationship between current speed and gap passages was shown for the impact treatment (*R* ^2^=0.509, *F*_1, 18_=18.646, *P*<0.001) but not for the controls (*F*_1, 18_=0.956, *P*=0.341), indicating an interaction effect between treatment and current speed. 

### Influence of visibility

For visibility, gap passages were positively related to visibility, regardless of rotor presence (impact: *R* ^2^=0.357, *F*_1, 18_=10.001, *P*=0.005; control: *R* ^2^=0.652, *F*_1, 18_=33.671, *P*<0.001). However, no correlation was shown (*F*_1, 18_=0.457, *P*=0.500) between visibility and the distance between fish and rotor (based on all fish taxa in samples with present rotor).

### Effects on fish assemblage

The genus level assemblage composition of fish passing through the gap differed between control and impact treatment (ANOSIM, *R*=0.318, *P*=0.001). The SIMPER analysis showed that many genera contributed to this dissimilarity and most taxa showed reduced numbers of passages when the rotor was present (Table 2). Although this indicates a rather general effect of the rotor among taxa, a significant deterrent effect was only shown for a few taxa. For the 17 tested fish genera (cumulatively contributing to 90% of the total dissimilarity in assemblage composition) univariate tests turned out significant for five genera when considering the whole span of current speeds (Table 2). A slightly different result, also with five significantly affected genera, was obtained when restricting the analysis to samples with higher current speeds (>0.6 ms^-1^). All these evidently affected genera were browsers, and most had a compressiform body shape. For all other tested genera, with no shown deterrence effect of the rotor, estimations of statistical power turned out low (<0.80). Therefore, non-significant results are not to be interpreted as ‘no effect’ for any fish genera.

**Table 2:** Detailed results showing effects on gap passages for fish genera contributing to most of the dissimilarity between control and impact treatment.

**Genus**	**Feeding guild**	**Body shape**	**Swimming style**	***D* (%)**	**∑ control (A)**	**∑ impact (A)**	**P (A)**	**∑ control (B)**	**∑ impact (B)**	**P (B)**
*Acanthurus*	Browsers	Compressiform	Carangiform	14	190	68	**0.000**	91	5	**0.000**
*Chaetodon*	Browsers	Compressiform	Carangiform	12	142	50	**0.005**	79	12	**0.011**
*Rhabdosargus*	Inv. feeders	Fusiform	Carangiform	10	125	71	0.989	101	57	0.912
*Ctenochaetus*	Browsers	Compressiform	Carangiform	9	131	43	**0.006**	70	18	0.052
*Siganus*	Browsers	Compressiform	Carangiform	8	95	6	**0.000**	57	0	**0.000**
*Thalassoma*	Inv. feeders	Fusiform	Labriform	8	113	78	0.478	85	31	**0.019**
*Scarus*	Browsers	Fusiform	Subcarangiform	7	93	17	**0.000**	53	6	**0.015**
*Sufflamen*	Inv. feeders	Compressiform	Balistiform	3	17	25	0.191	14	11	0.853
*Centropyge*	Browsers	Compressiform	Carangiform	3	32	3	0.277	1	0	0.739
*Kyphosus*	Browsers	Fusiform	Subcarangiform	3	31	1	0.265	0	0	-
*Plectorhinchus*	Inv./fish feeders	Fusiform	Subcarangiform	3	25	11	0.341	18	7	0.353
*Lethrinus*	Inv./fish feeders	Fusiform	Carangiform	2	24	19	0.620	11	10	0.739
*Pomacanthus*	Browsers	Compressiform	Carangiform	2	18	7	0.192	4	0	0.739
*Lutjanus*	Inv./fish feeders	Fusiform	Carangiform	2	16	1	0.174	8	1	0.247
*Parupeneus*	Inv. feeders	Fusiform	Subcarangiform	2	13	3	0.072	7	0	**0.007**
*Bodianus*	Inv. feeders	Fusiform	Labriform	1	14	8	0.512	11	6	0.529
*Scolopsis*	Inv. feeders	Fusiform	Carangiform	1	6	11	0.738	2	1	0.739

The first columns indicate the taxonomic identity and categories of fish. The genera-specific contribution to the assemblage dissimilarity between fish passing through the gap during control (no rotor) and impact (rotor) is indicated by *D*. Total numbers of gap passages and significance values (*P*) for effects of the rotor (Mann–Whitney *U* tests, using 2×1-sided exact *P*) are presented separately for (A) all samples (*n*=20) and for (B) samples in current speeds above 0.6 ms^-1^ (*n*=10). Significant effects are indicated in bold. All non-significant results were associated with low power (<0.8). Only fish genera cumulatively contributing to 90% of the assemblage difference are shown in the table.

### Rotor avoidance zone

**Figure 3:**
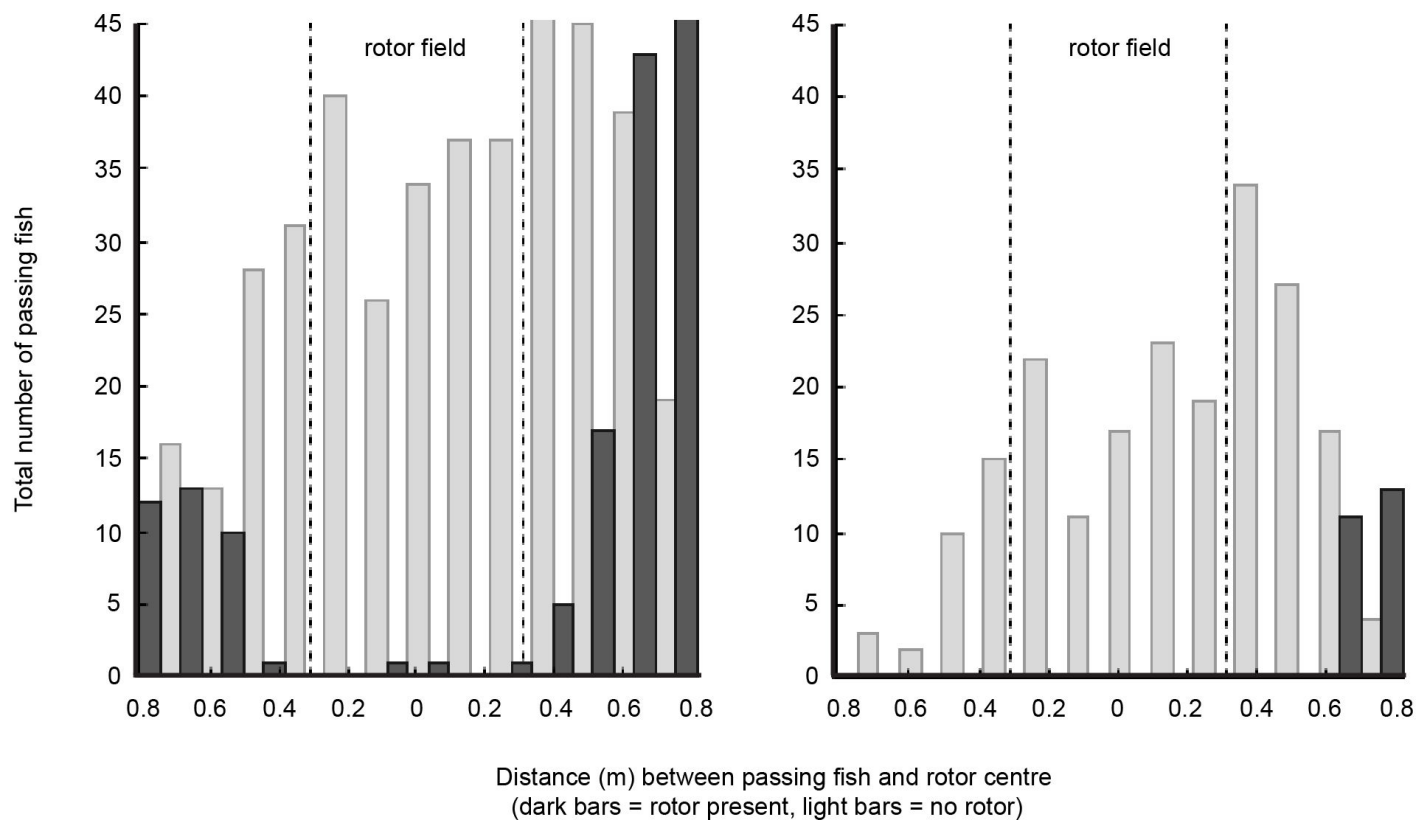
Distance between fish and rotor for all samples (left) and strong currents only (right). The histograms show the total number of measured fish passing through the gap at different distances from the rotor centre. The distance, given in metres, represents the closest range between fish and the central rotor spindle. Dark bars show samples with the rotor in place (impact treatment) and light bars represent samples without rotor (control). The edge of the rotor is indicated by dotted lines. For example, 0.6 m distance from the rotor centre corresponds to approximately 0.3 m from the rotor edge. The left panel includes all samples of the study and the right panel exclusively includes samples with current speeds above 0.6 ms^-1^. Only fish swimming within the camera stereo-field are included in the diagram (i.e. fish passing through the gap by swimming close to the fringing rocks could be counted but not measured and are therefore not shown).

In order to establish the range of avoidance from the rotor, the distance between passing fish and the rotor field was investigated. For the observed reef fish, a generic avoidance zone extending about 0.3 m from the rotor edges is indicated by Figure 3. No significant relationship was found between passing distance and total length of fish (ANOVA; *F*_1, 186_=2.418, *P*=0.122). However, in the presence of the rotor a difference in passing distance was shown among the most common fish genera (*Acanthurus*, *Rhabdosargus*, *Thalassoma*, *Chaetodon* and *Ctenochaetus*) (Kruskal–Wallis test, X^2^=21.207, d.f.=4, *P*<0.001). Stumpnoses (*Rhabdosargus* spp.) passed significantly (*P*<0.05) closer to the rotor than each of the other genera, with some individuals moving as close as 10 cm from the rotor.

**Figure 4:**
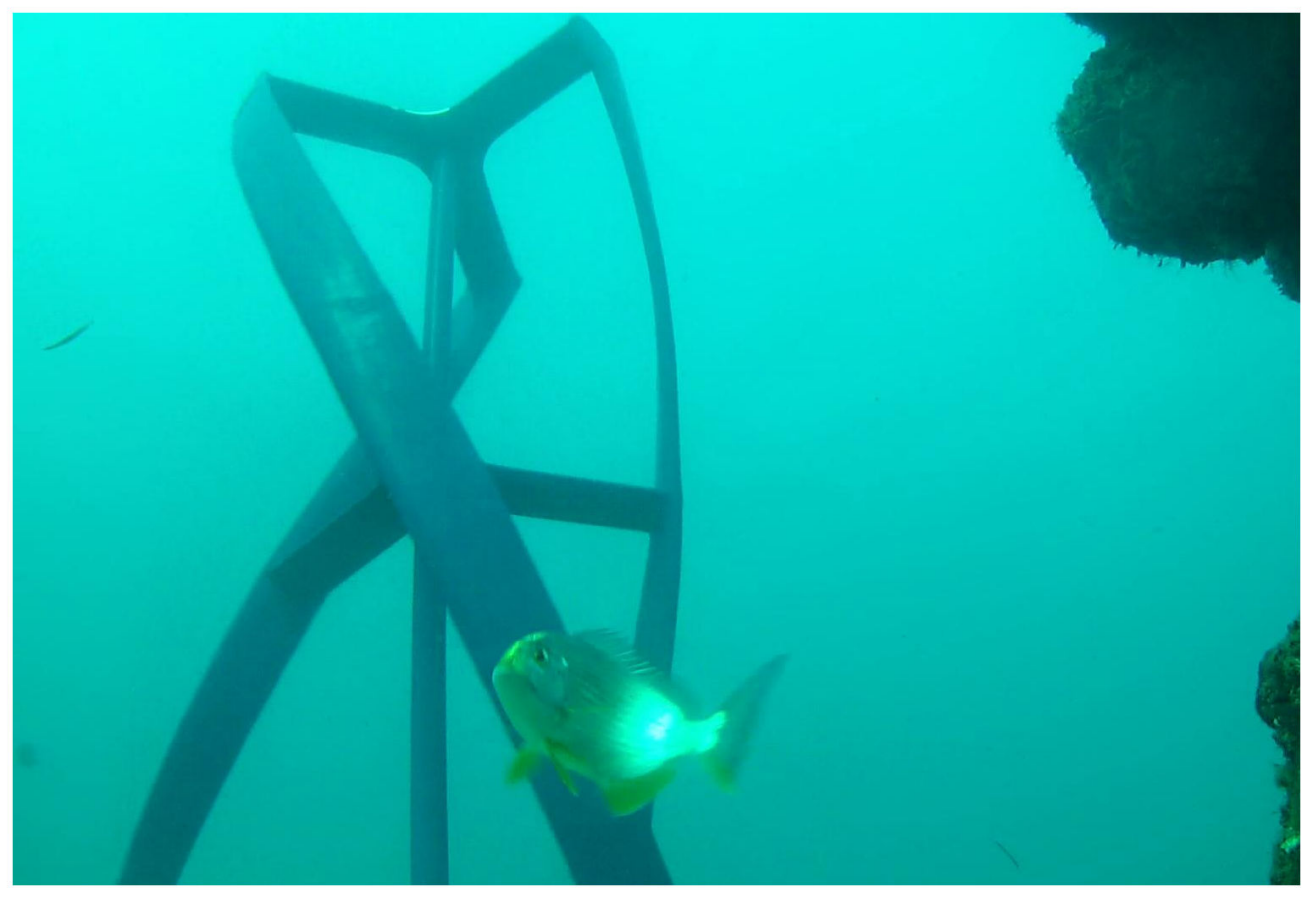
Example of *Rhabdosargus sarba* (F.) evasion manoeuvre. Goldline stumpnose *R. sarba* carrying out a typical evasion manoeuvre as the specimen passes through the gap against a 0.7 ms^-1^ current speed. The fish changed its trajectory 45° with a quick burst as it was startled by the approaching rotor blade at 22 cm distance. The image was extracted from the analysed video material (right camera).

**Figure 5:**
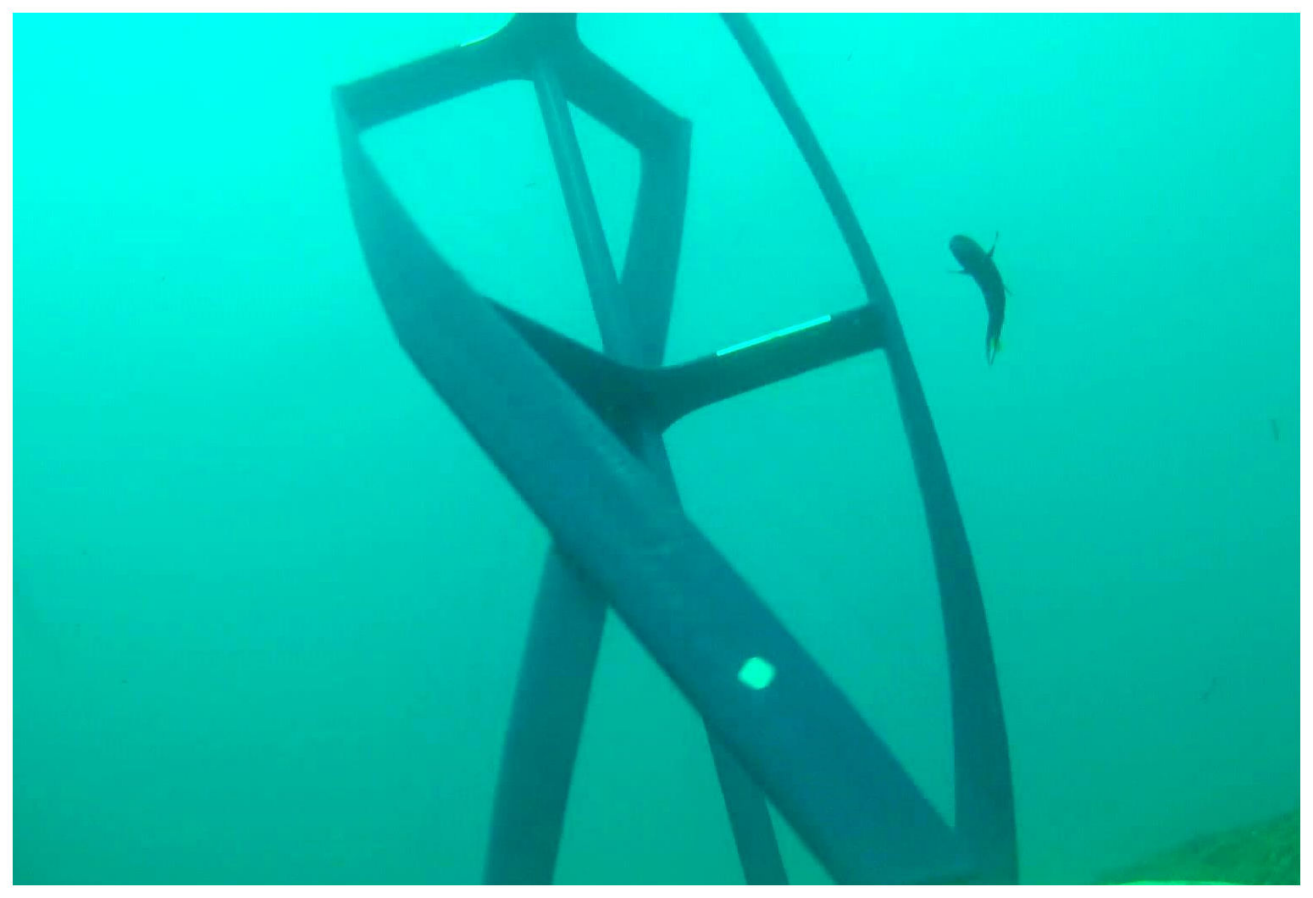
Example of *Thalassoma lunare* (L.) evasion manoeuvre. Moon wrasse *T. lunare* responding to the approaching rotor blade by performing an agile move around the blade and continuing its chosen trajectory through the gap. The closest distance between this specimen and the rotor was 12 cm, and the interaction took place in 0.7 ms^-1^ current speed, with the fish swimming against the current. The image was extracted from the analysed video material (left camera).

Only 19 specimens, from six genera, performed distinct evasion manoeuvres when approaching the rotor. The evasive manoeuvres were characterized as a startle at a mean of 27.0±2.5 (SE) cm from the rotor edge. Stumpnoses (*Rhabdosargus* spp.) and wrasses from the *Thalassoma* genus performed most of the evasions and no significant differences in startle distance were found between the two kinds of fish (ANOVA; *F*_1, 11_=1.249, *P*=0.288). For the stumpnoses, all evasion manoeuvres took place in current speeds above 0.60 ms^-1^ and were, as for most taxa, characterized by a distinct turn (45–90°) and burst swimming away from the rotor (Figure 4). By contrast, the evasion manoeuvres of the wrasses were generally characterized by agile moves around the rotor blade (Figure 5).

**Figure 6:**
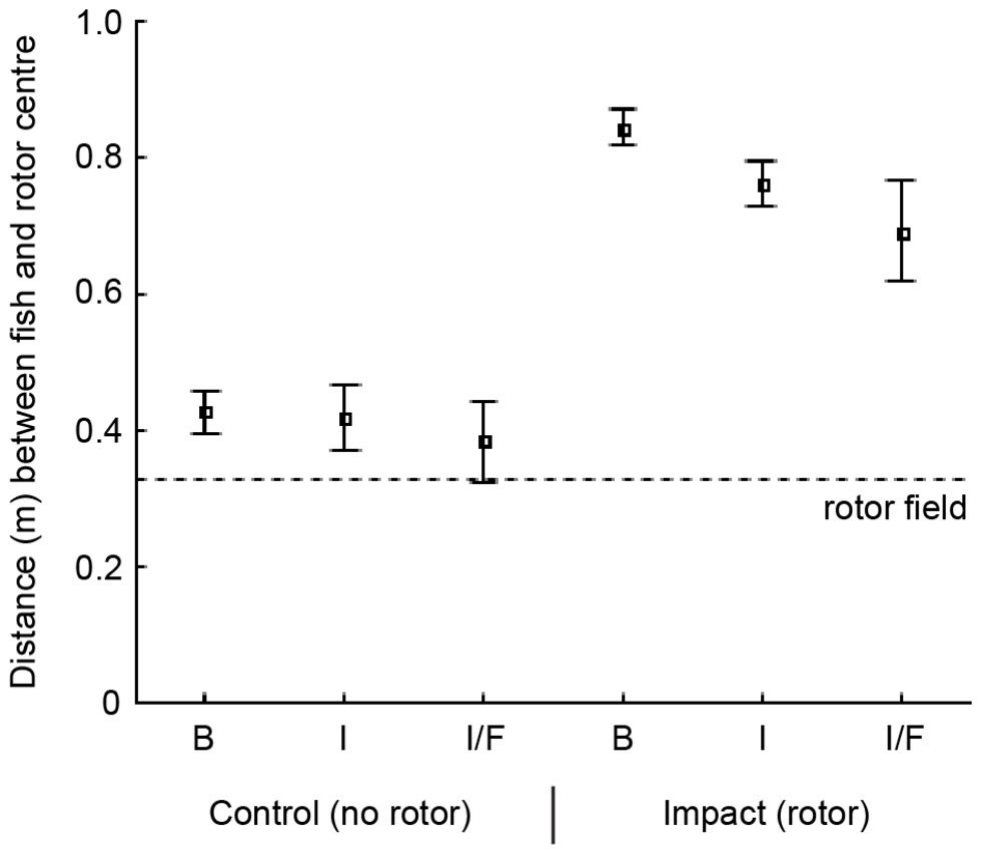
Distance between fish and rotor for feeding guild categories of fish. Means and 95 % confidence intervals of the closest distance (metres) between passing fish and rotor centre, during control conditions and in the presence of the rotor. The dotted line indicates the outer edge of the rotor. Only fish observed within the camera stereo-field are included. Feeding guilds: **B** browsers (control *n*=243, impact *n*=121), **I** invertebrate feeders (control *n*=162, impact *n*=128) and **I/F** invertebrate/fish feeders (control *n*=54, impact *n*=19).

In absence of the rotor no differences in passing distance were shown among feeding guild categories of fish (browsers, invertebrate feeders and fish/invertebrate feeders). But with the rotor in place there was a significant difference among groups (Kruskal-Wallis test, X^2^=19.827, d.f.=2, *P*<0.001) where browsers (mainly herbivores) kept a greater distance from the rotor edge than invertebrate feeders (*P*<0.001) and fish/invertebrate feeders (*P*=0.002) (Figure 6).

**Figure 7:**
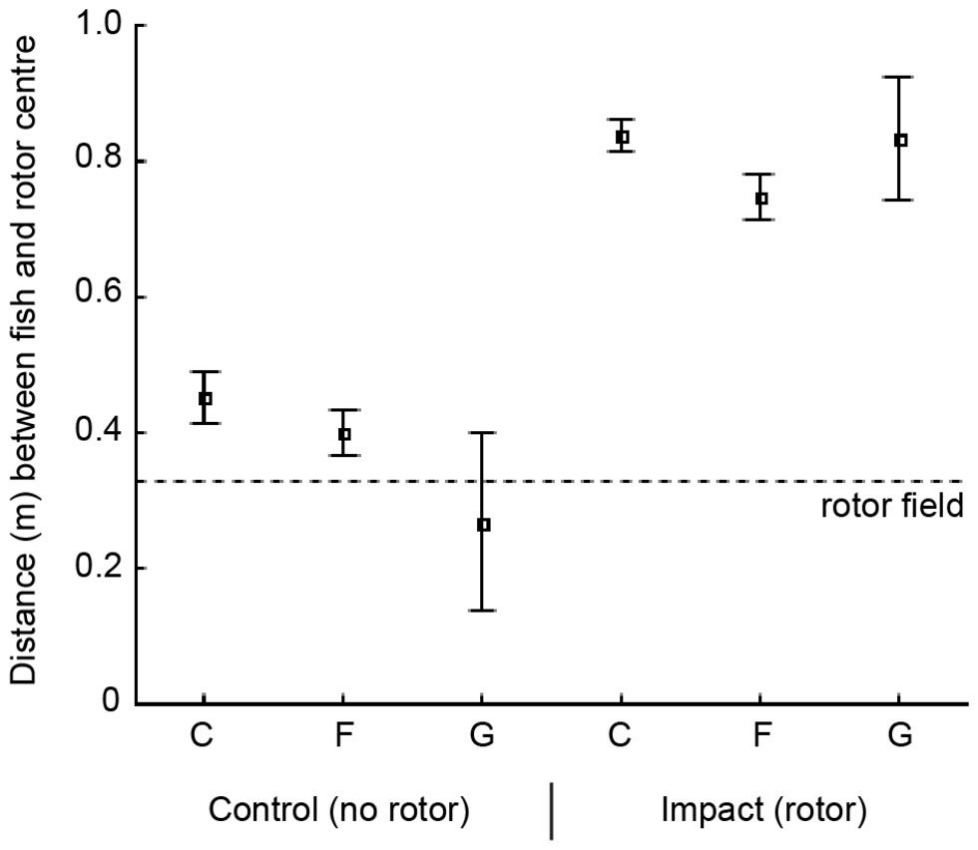
Distance between fish and rotor for body shape categories of fish. Means and 95 % confidence intervals of the closest distance (metres) between passing fish and rotor centre, during control conditions and in the presence of the rotor. The dotted line indicates the outer edge of the rotor. Only fish observed within the camera stereo-field are included. Body shapes: **C** compressiform (control *n*=201, impact *n*=129), **F** fusiform (control *n*=249, impact *n*=134) and **G** globiform (control *n*=9, impact *n*=5).

Among the three different body shape categories (fusiform, compressiform and globiform) the passing distance differed significantly both during controls (Kruskal–Wallis test, X^2^=7.988, d.f.=2, *P*=0.021) and when the rotor was in place (Kruskal–Wallis test, X^2^=15.961, d.f.=2, *P*<0.001). Compressiform fish passed through the gap at the farthest distance from the rotor field both with and without the rotor in place (Figure 7), but the post hoc test only turned out significant (*P*<0.001) for the difference between compressiform and fusiform fish in presence of the rotor. No significant differences in passing distance were found among fish swimming types (Kruskal–Wallis test, X^2^=1.417, d.f.=4, *P*=0.841).

In respect to effects on large predatory fish, the distance between approaching kingfishes (*Caranx* spp.) and the rotor field was clearly greater during rotor presence (ANOVA; *F*_1, 18_=14.421, *P*=0.001). That is, during controls the fish moved closer to where the rotor edge would have been than they did when the rotor was actually in place. While kingfish individuals were observed to pass through the gap when the rotor was absent, no specimens moved closer than 1.1 m from the edge of the rotor. In samples with current speeds above 0.6 ms^-1^ the closest distance to the rotor edge was increased to 1.7 m. Also, barracudas (*Sphyraena* spp.) were frequently observed in the area, but most often too close to the surface to allow for measurements.

## Discussion

### Main findings

Contrary to our first hypothesis, the rotor did not prove hazardous to fish during the tested daylight conditions. However, in accordance with the other tested hypotheses, it was shown that the presence of the rotor had a deterrent effect on fish, with differences among taxa. As predicted, the magnitude of the deterrent effect was correlated with tidal current speed. 

Our results, confined to the studied vertical-axis turbine and moderate current speeds, support the sparse evidence from other studies [13-15], that fish are able to avoid collision with open flow hydrokinetic turbines during daylight conditions. Out of all observed fish only two specimens of bluestreak cleaner wrasse (*L. dimidiatus*) entered the rotor, during low current and low rotational speeds. The bluestreak cleaner wrasse is a particularly bold species that functions as cleaner of larger clients, including predators [51]. Other fish consistently adjusted their swimming patterns to avoid close encounters. Thus, the rotor both had a deterrent effect and an avoidance zone exceeding the rotor diameter (generalized to about 0.3 m for reef fish). Neither the deterrent effect nor the avoidance zone have previously been established.

### Influence of environmental variables

Increased current speed had a negative effect on gap passages in the presence of the rotor, with fewer fish passing through the gap as water velocity increased. No similar pattern was shown during control conditions, although fish are known to seek shelter when the current increases [12,52]. Consequently, high current speed enhances the deterrent effect of the rotor, independently of natural effects of current speed. This result complies with previous observations [13] and could be explained either by lowered fish manoeuvrability or increased rotational speed influencing the deterrent effect of the rotor, or a combination.

The number of gap passages was positively related to visibility, both when the rotor was in place and during controls, indicating a general reduction of fish movements in the area as turbidity increased. Visibility was not shown to influence the distance that fish kept from the rotor, but even in the most turbid samples visibility was at least a few meters.

### Different response for different groups of fish

Different taxonomic groups showed varied responses to the rotor. Surgeonfish (*Acanthurus* spp. and *Ctenochaetus* spp.), butterflyfish (*Chaetodon* spp.), rabbitfish (*Siganus* spp.) and parrotfish (*Scarus* spp.) were the most evidently deterred benthic fish, with significantly fewer gap passages in the presence of the rotor. These genera are all browsers, and all but parrotfish have a compressiform body shape.

It was shown that browsers kept a greater passing distance from the rotor compared to other feeding guilds. One interpretation would be that the mainly herbivorous browsers are more cautious of the rotor than fish at a higher trophic level, but such a hypothesis remains doubtful and lacks support from previous work on fish boldness [35]. Considering that most browsers are compressiform, it is also possible that an explanation of the observed pattern lay in fish body shape. In strong currents the compressiform body shape, with its large vertical surface, may be more difficult to manoeuvre than the slender fusiform shape [53]. Compressiform fish moved through the gap at a greater distance from the rotor field than fusiform fish, regardless of treatment, possibly reflecting a habit of avoiding the centre of the gap where water velocity is highest. With the presence of the rotor, the difference between these two body shape groups increased. Although compressiform fish have a high manoeuvrability in ordinary conditions [27], the current and turbulence may have increased the preferred safety distance from the perceived hazard to a larger extent than among less restricted fusiform fish. The performance of globiform fish could not be further investigated because few specimens possessing this body shape were observed.

Interestingly, very few fish used burst speed evasions to avoid the rotor. This may indicate that fish generally detected, and remained aware of, the rotor and that maximum swimming speed capacity was of little importance for avoiding the relatively small rotor under test.

None of the studied fish genera was clearly unaffected by the rotor (non-significant results were of low statistical power). However, stumpnoses (*Rhabdosargus* spp.) passed the rotor at a significantly closer distance than other fish genera. These fish have a sturdy appearance but are laterally compressed for having a fusiform shape [54], and one specimen even collided with the rotor foundation as it was struggling in the current. Thus, it is possible that a lower response among stumpnoses is related to bold behaviour. Other less affected fish frequently observed to perform agile manoeuvres around the rotor were wrasses from the *Thalassoma* genus, recognized for their particular inquisitiveness [55]. In conclusion, interspecific differences among reef fish in response to the rotor might be explained both by morphological (body shape) and behavioural (boldness) traits.

For large predatory fish the results showed that kingfishes (*Caranx* spp.) avoided the rotor at much greater distance than smaller reef fish, possibly reflecting the previously observed pattern that cautiousness increases with size among many fish species [55], as large fish generally have lower acceleration [29] and manoeuvrability [27].

### Limitations of the study

Importantly, this study is confined to effects of the rotor during daylight. At night, fish will have reduced possibility of detecting a rotor by visual senses and collision risk may increase. A reduced reaction distance for fish approaching hydrokinetic turbine rotor blades during the night as compared to the daytime has previously been reported [13]. Fish assemblage composition and spatial distribution of fish differ between night and day, and many of the species in this study are strictly diurnal [56]. Hence studies under dark conditions, and with adapted equipment [57], should be performed. 

The colour of the rotor (midnight blue) is likely to have influenced the results. Among fish, it is often the brightness contrast that determines detection of objects, and a colouration that is different from the ambient colour is more easily detected [58]. A brightly coloured or fluorescent rotor would therefore be more easily detected in the turbid coastal water conditions of this study, probably generating a stronger deterrence effect, while for instance a red-coloured rotor would be less visible due to the spectral transmission properties of seawater [59].

### Implications of the study

This study implies that vertical-axis turbines of the dimensions tested here are not hazardous to fish during daylight conditions and the tested current speeds. Although the level of deterrence and the spatial extent of rotor avoidance differed among different taxa no fish appeared close to collision. Choosing a more contrasting colour of the rotor would probably further reduce any collision risk (the most suitable colour for the purpose would vary according to locations and depths [59]). The study was confined to moderate current speeds, but results indicate that higher current speed could increase the level of deterrence rather than increasing the risk of collision. Importantly, large predators – associated with high ecological value [60] – showed particularly high cautiousness and should therefore be at low collision risk.

Nevertheless, the deterrent effect of the rotor was profound. Several of the significantly deterred genera are widely distributed herbivores of particular importance for controlling algal growth on reefs (surgeonfish, rabbitfish and parrotfish) and the apparently most affected taxa, kingfishes, represent important apex predators [61]. Species from all these genera are dependent on a number of habitats during different life stages and often migrate among habitats over the tidal and diurnal cycles [62]. In this study, fish movements were never fully restricted by the rotor, as those avoiding the gap could swim around the surrounding rock formations, but large installations of multiple turbine systems could create selective barriers across tidal straits. So large turbine installations may impede habitat connectivity by restricting migration patterns and decreasing the ability of functional groups to perform their roles in the seascape [63].

The generalized rotor avoidance zone was about 0.3 m for smaller reef fish that passed by the rotor while large predatory fish did not approach the rotor closer than 1.7 m when currents were strong. While a partial restriction of fish movements at a turbine site is not likely to have ecological implications, the findings of this study suggest that systems with multiple turbines should be designed to leave at least one metre of free space around rotors to allow reef fish to pass through, and several metres space to make sure that large predators can pass through. At a more detailed level, technical design of multiple turbine systems can be guided by knowledge of local fish fauna and the findings from this study on the role of morphological and behavioural traits for fish response to the rotor. It should be noted though that extrapolation of detailed behavioural responses to taxa not studied here will be uncertain [20]. Although the findings of this study provide one step towards alignment of marine conservation and ocean energy interests, further research is important. In particular, the effects of hydrokinetic turbines during low light conditions need to be addressed.
